# Transition to Adulthood for Children Who Experienced Acquired Brain Injury: Lessons Learned from a Clinical Program for Children and Adolescents

**DOI:** 10.3390/children13081086

**Published:** 2026-08-16

**Authors:** Juliet Haarbauer-Krupa, Lyn Turkstra, Peter Meulenbroek, Mimi Gold, Jason Amos

**Affiliations:** 1 Department of Pediatrics, Emory University School of Medicine, Atlanta, GA 30322, USA; 2 School of Rehabilitation Science, McMaster University, Hamilton, ON L8S 4L8, Canada; 3 Department of Communication Sciences and Disorders, Radford University, Radford, VA 24142, USA; 4 Rehabilitation Services, Children’s Healthcare of Atlanta, Atlanta, GA 30342, USA

**Keywords:** acquired brain injury (ABI), traumatic brain injury (TBI), pediatrics, transition to adulthood, healthcare transition, career transition

## Abstract

**Highlights:**

**What are the main findings?**

- This manuscript details what was learned from a program to address the transition to adulthood for adolescents who experienced an acquired brain injury.
- We are examining the effects of social and environmental factors on health and development as well as those dealing with clinical issues and the organization of services.

**What are implications of the main findings?**

- Adolescents who experience a childhood ABI benefit from education about the injury effects, technology use for self-management, peer coaches and connection to both healthcare and career opportunities following high school graduation.
- It is important to address both healthcare and school/career transition.

**Abstract:**

**Background**: The purpose of this paper is to describe lessons learned from a clinical program that was developed to serve adolescents who experienced ABI in childhood during their transition to adulthood by providing insights to achieve good health and assessment of needs in both healthcare and educational/career areas. **Methods**: Participatory Action Research methods were utilized for program development by collaborating with focus groups of teen participants and stakeholders following each year. Activities were devised based on best practices for cognitive rehabilitation, direct instruction, and practice of cognitive and metacognitive strategies, technology use, peer coaches and incorporation of multiple stakeholders, including family members. **Results**: We learned the importance of technology utilization, completing surveys of vocational interests, peer coaches, and post program follow-up to better understand both healthcare and career needs of adolescents who experienced TBI and are transitioning to adulthood. **Conclusions**: Adolescents who experience a childhood ABI benefit from education about the injury effects, technology use for self-management, peer coaches and connection to both healthcare and career opportunities following high school graduation.

## 1. Introduction

Children and youth can sustain an acquired brain injury (ABI) at any time in their development. The most common cause of childhood ABI is traumatic brain injury (TBI), but children also can sustain ABI from etiologies such as brain cancer [1]. If the injury is severe enough to need immediate medical attention, children and youth are most likely to access medical services through the emergency department (ED) and receive follow-up from their pediatrician or family physician [2]. Those who sustain mild injuries may visit the ED or their pediatrician, or may not seek medical treatment at all, despite growing awareness that these injuries may have long-term consequences [3]. For an ABI of any severity, only a fraction of all levels of brain injuries is known to the community and school systems, who are increasingly the sole provider of care for those with long-term disabilities [4].

Childhood ABI can disrupt new and emerging skills that are critical for academic functioning during the school years, and can have a profound negative influence on education, employment, psychosocial, and community outcomes following high school [1,2,5,6]. Although there is limited information about adult outcomes for children who experience ABI, a consistent emerging theme is that these children are not getting the long-term services and supports needed [5,6,7,8]. Further, recent studies indicate that children who experience an ABI during early (ages 0–5 years) or middle childhood (ages 6–18) demonstrate worse outcomes, particularly in executive function, which impacts socialization and school performance. Children can also experience persistent symptoms which may show up later in development [9,10,11,12]. We do not have a clear understanding of how this acquired chronic health condition affects the attainment of adult milestones such as high school graduation, enrollment in post-secondary education, and obtaining employment [1].

A critical stage for youth is the transition to adulthood, a move from dependence to independence, which begins during the high school year [13]. These years are a critical stage in development when youth are faced with both opportunities and constraints. For high school students, the transition to adulthood begins in early high school and continues into at least the late 20s [14,15]. Students with disabilities can face more obstacles and barriers than their uninjured peers when preparing for or during the transition [16]. Disability severity and access to and availability of resources contribute to transition outcomes, resulting in differential outcomes for post high school education, employment and independent living [17]. Children with developmental disabilities enrolled in special education programs under the Individuals with Disability Act (IDEA) or a 504 plan (under the American with Disability Act (ADA) and Section 504 of the Vocational Rehabilitation Act) have transition plans created for them beginning at age 14 or when they enter high school as a part of their educational program (US Department of Education). However, those who are not identified at school or those who do not qualify for special education do not have a transition plan.

In addition to planning for educational and vocational outcomes, childhood survivors of ABI move from pediatric to adult medical providers. For children with medical conditions, a primary factor for optimal transition is the presence of a medical home, defined as “an approach to providing comprehensive and high-quality primary care” [18]. Having a medical home is associated with higher satisfaction with care and fewer unmet healthcare needs [19], and increases the likelihood of a successful transition to adult services [20]. Adolescents’ understanding of their medical needs, healthcare self-management responsibilities, and greater satisfaction with their care contribute to better health and wellness following the transition to adult care [21].

By the time they graduate from high school, typical adolescents have become less reliant on adults, have more independent decision-making ability and responsibilities, and are increasingly choosing their social, school, work, and community activities rather than having them chosen by parents or dictated by school. Community participation is individually established based on interest and access to resources. A childhood ABI can change any or all outcomes. Teens and their families can be dealing with challenges they never expected, such as cognitive and behavioral changes, socialization, community participation, and adjustment to a catastrophic injury, any of which can alter future plans and hopes for the future. Transition support needs become more immediate and complex, and access to these supports can make a critical difference in who continues to postsecondary programs [7].

While we know that children and youth with ABI need a medical home that includes a primary care provider and support for transition planning, few providers outside of medical settings can address both needs. School systems offer transitional plans as part of an Individualized Education Plan (IEP) for those enrolled in special education; however, they are not geared to the unexpected challenges and adjustments for teens with ABI. Pediatric medical systems of care are rarely aligned with adult models of care to support the transition. Further, teens and their families may not be aware of long-term consequences of an ABI experienced as a child. Teens with ABI and their families are left to navigate both models of service with little support for the transition to post-high school life.

Transition readiness is a critical factor for supporting positive outcomes for adolescents and young adults [2,22,23,24,25,26,27,28]. A key component of this process is adolescent responsibility and autonomy related to health-care management [17,28]. To date, no identified programs that address both healthcare and career transitions for teens who experienced any ABI during childhood have been developed. The purpose of this paper is to describe lessons learned from establishing a model program in the healthcare system that was developed to serve adolescents, who experienced ABI in childhood and were transitioning to adulthood by providing insights to how to achieve good health, assessment of needs in both healthcare and educational/career areas and learn from the program.

## 2. Materials and Methods

### 2.1. Program Development

The study was conducted in accordance with the Declaration of Helsinki, and we received IRB approval for program development (Children’s Healthcare of Atlanta IRB 10-0173), so will be reporting on what we learned clinically from the program. Specific information about participants was not included in this IRB. All activity was determined to be program development when submitted to the IRB at Children’s Healthcare of Atlanta.

The program was named program participants and stakeholders as the B.R.A.I.N Program (*Bringing Rehabilitation and Injury Recovery to New Levels*). The first year of the program was supported by pilot funding from Children’s Healthcare of Atlanta. Future years were funded by a variety of foundations. Participants were not assessed program costs. All activity was determined to be program development when submitted to the IRB at Children’s Healthcare of Atlanta.

To develop the program, we used Participatory Action Research methods, collaborating with focus groups of teen participants and stakeholders (parents, physicians, educators, state agency personnel) [29]. We based activities on best practices for cognitive rehabilitation for children and youth, including use of personalized goal setting [30], direct instruction and practice of cognitive and metacognitive strategies [31], and incorporation of multiple stakeholders, including family members [32].

In the first year, the program focused on cognitive rehabilitation and strategies to improve learning. In focus groups at the end of that session, teens and their families shared that they wanted more than a program that focused on academic learning strategies. They specifically requested the addition of a fitness portion and a mechanism to determine leisure activity preferences. Thus, in the second year the program model expanded to provide a greater focus on self-determination, emphasizing goal setting and achievement of individualized short-term objectives to meet post-high-school goals.

### 2.2. The B.R.A.I.N. Program: Conceptualization and Development

Our clinical experience working with adolescents with ABI, combined with the research literature, indicated that few adolescents were receiving transition services geared to their injury needs. From parent report, we learned that many were tracked into a “life-skills” curriculum that terminated in an exit with a modified diploma, some had not received any specific transition activities, and many were denied transition services through the schools until their senior year. Some were set to graduate on time with their class, which meant abrupt discontinuation of their education-based services, with no plans for community services. The focus of the school system was typically on preparation to graduate on time, without development of a linkage to community-based supports and training in independent living skills. This experience, and the research findings summarized earlier, led to the conception of a summer program for teens with ABI which started in 2009 with two participants and expanded to ten participants each year between 2010 and 2015. The program aimed to provide an opportunity for teens to return to a medically based rehabilitation model to determine if medical, education, vocational, and community participation pathways for transition had been established. We chose the summer when school was not in session so students could participate full-time in the program.

### 2.3. Program Structure

Participants received a pre-program assessment which included a parent report of their child’s history (i.e., age, parent education and employment, type of insurance, post-injury follow-up in a healthcare or school setting), parent report of time management, self-report of time management, vocational interest inventory [33], leisure assessment and the Functional Independence Measure [34]. This information was used to better understand participants entering the program. Participants attended the program daily from 9 a.m. to 3 p.m. for two weeks. The program was designed to address skills that contribute to successful transition including self-determination (goal setting, communication, and self-management) and health and wellness (identifying a medical home, health self-management, and fitness). Sean Covey’s book, *7 Habits of Highly Effective Teens*, was used to generate discussions. We provided education on returning to driving following an ABI, vocational consultation (including a vocational interest inventory) and assessment of leisure preferences.

On the first day of the program, participants completed a positive psychology survey to determine their strengths and reported the findings to the group [35]. We hired college students to serve as peer coaches for the program. The coaches were undergraduates who majored in rehabilitation areas (physical, occupational or speech), education, psychology or pre-med. Prior to the first day of the program, the coaches received half day, in person training on program structure, goal setting, and effective communication skills conducted by the lead author. Each peer coach was matched 1:1 with program participants. Program components are described in Table 1.

Two research collaborations contributed to the program: (1) a research pilot study by Professor Frank Durso, PhD and students from a local university, which tested the use of cell phone technology at the time as a cognitive prosthesis and funded the purchase of iPod Touch devices, technology that was new at the time; and (2) a collaborative research project with Professor Lyn Turkstra from the University of Wisconsin Madison to devise communication skills coaching for participants. As part of this collaboration, peer coaches were hired and trained to support participant communication skills. We followed-up with program participants via telephone at 3 and 6 months. We asked participants and their parents to complete satisfaction surveys on the last day of the program. Progress in each area was tracked to determine individual and group improvements for program participation.

### 2.4. Program Activities

The program included activities for health and wellness, including a fitness assessment and focus on self-management of health. Thus, the program provided a combination of education, time management, fitness and wellness, and communication skills. We provided teens with strategies for improving health and academic performance, determining career goals and interests, and reviewing their existing educational programs. We provided participants and their families with information on topics such as driving rehabilitation, returning to competitive sports, and planning for transition to adulthood. When we learned that all the teens owned cell phones, we incorporated their phones into strategies for time management. Participants met with peer coaches about the best way to use their cell phones to keep track of assignments and program goals.

## 3. Results

### 3.1. What We Learned from the B.R.A.I.N Program

For each year of the program, enrollment was ten teens between ages 14 to 22 years who were at least 6 months post ABI and living in their home communities and experienced a head injury during childhood prior to age 18. Over 5 years that we offered the program, we served 50 participants. Those from rural areas stayed locally at the Ronald McDonald House, the Manley House, or with friends in the area. Participants goals included attending college, driving, and participating in athletics. Program outcomes included providing recommendations for IEP revisions and development of transition plans, links to services and consults for sports and community participation, and education on the rehabilitation driving process, risk for sports-related concussions, and students’ and parents’ rights in IEP meetings. Participants also learned to use their cell phones for time management, alerts and reminders.

Following the program each year, participants and their parents were asked to complete a survey following the program which offered a Likert rating scale and space for comments. Each time, program satisfaction was consistently rated as excellent by the parents who stated that BRAIN gave their teens more social confidence while helping them establish potential career paths. They also indicated this program provided an opportunity to meet with other parents and specialists/staff helped in getting their child back in school. Related to their child, parents reported that they see a breakdown after high school in the transition to college and jobs. While BRAIN helped teens establish social confidence and identify interests, reaching these academic and career goals has continued to present a barrier for parents and teens. The participants rated the program as “good” and reported that the program required demanding “schoolwork” rather than simply projects. When teens discuss their current challenges, they tend to focus on physical and cognitive issues related to their injury, such as difficulty walking, apraxia, and slower processing and communication. Overall, teens felt BRAIN offered a space to socialize in a safe environment, and they liked the ability to meet other people their age who have experienced TBI. Teens also stated that BRAIN provided education on how they might advocate for themselves and receive academic services, and it allowed them to explore what career opportunities may be available to them after school. Teen and parent respondents noted that being around other teens with TBI who had similar types of goals also made participants more motivated in returning to school. However, while respondents noted that being in BRAIN and surrounded by peers and supportive staff helped at the time, especially in terms of social and moral support, they experience continued difficulty in carryover to independent goal setting for school and employment.

Stakeholder group feedback overall indicated that providing options to cover program costs, adding content and activities for fitness and wellness, seeking a larger space, and including teens with ABI who have similar sequelae were important aspects of the program. We also learned that improvement was needed to better coordinate program arrival and departure as well as pre- and post-follow-up to make sure participants received recommended services. Participants suggested to offer the program later in the day to accommodate teen’s summer schedules.

### 3.2. Medical Home

During the first 5 years of the program, about two-thirds of participants and their caregivers identified a pediatrician or family practitioner as their medical home. The remaining participants either used community health (22%) or did not identify a medical home due to lack of health insurance of any kind (11%). All who identified a medical home reported seeking referrals for rehabilitation medicine follow-up care for their injury from this source.

### 3.3. Goal Setting

Through program activities, most participants (88%) were able to set long-term goals for post-high school plans by indicating a preference for attending college or a post-secondary program or plans to seek employment. Participants tracked goals for communication, school, fitness and time management using Goal Attainment scaling. At the end of the program, we asked participants if they achieved their own goals. The best most consistent improvement was in fitness. Participants were able to track the number of exercises or time spent on a treadmill achieved in their individualized program. Participants did not show as much improvement using this method for communication goals, time management, or writing goals in the school portion. We learned that these areas were more difficult for participants to operationalize.

### 3.4. Use of Cell Phone Technology for Self-Management

Following the program, many participants continue to use the iPod Touch for internet access, downloading applications (Latin review, math fact review) and notes (Evernote). We found that all teens were interested in using the iPod touch but persisted with their own personal cell phones to record calendar events and set reminders. Some participants utilized a free voice outpoint app called “Locabulary” in the program, particularly in community outings, and continued to use this feature after the program. While in the program, participants expressed concern about using the iPod touch or cell phones to help them at school. Reasons identified were (1) school rules prohibit the use of cell phones during school time and (2) stigmatizing—they did not want to look different than their peers. In the months following the program, four of the teens sent text messages to the program coordinator with questions during school time, including “can you help me get into this class?”, “How do you transfer documents in Evernotes to Microsoft Word?”, “Can you help me with this college application?” and informing her that an iPod touch had been stolen from a backpack.

### 3.5. Communication Skills

As part of the program, peer coaches were hired from local colleges and received training on ABI and how to facilitate participant communication skills by utilizing four strategies: *Strategy 1: OWL-Observe* (become familiar with how and why the adolescent communicates) *Wait* (give time and encouragement to express ideas and convey expectations to communicate) *and Listen* (give undivided attention and encouragement to continue the conversation). Strategy 2: Talk do not test (give information and ask positive questions); Strategy 3: It takes two (expand and interpret what the adolescent say); and Strategy 4: Be a Supermodel (think out loud and be a “cognitive model” and provide opportunities for practice [38,40].

### 3.6. Educational

For students enrolled in special education (66% of participants) the program teacher reviewed the current IEP. Many of the IEPs did not reflect a current assessment of the participant’s skill level or contain a detailed transition plan. Two students were recent high school graduates and only one was connected to vocational rehabilitation. Since many students enrolled in the program had goals to attend college or were recent graduates of high school without enrollment of school, we focused on written skills in the educational component. For participants who were still in high school but not receiving educational supports, we worked with their school system to develop any needed supports identified in the program. Each participant also completed a vocational interest inventor (STRONG vocational interest inventory) and interview with staff to determine their interests. We learned that many participants had not indicated interests previously to their parents or school.

### 3.7. Fitness and Wellness

All participants improved from their initial baseline and received an individualized program for home use at discharge. We learned that some participants had returned to sports without consulting a physician and were experiencing difficulties. Several participants reported continued use of their fitness program and goal tracking as indicated by follow-up.

### 3.8. Program Follow-Up

We typically called families six months after to inquire about participants’ status. In 2015, we also reached out to all participants who participated in the program from 2010 to 2015. Although we did not collect specific data, we did learn, anecdotally, participant and family opinions about how program participation helped them. A sample of individual stories are listed in Table 2. Overall, participants appeared to benefit from program participation in at least one area that was described above.

## 4. Discussion

Transitioning to adulthood is a complex time for adolescents with brain injuries that requires understanding both healthcare and educational/career transition. Although programs such as the B.R.A.I.N program are not available everywhere, lessons learned from our program can inform how to improve outcomes for adolescents who experience ABI. Initial program aims were to ensure that those who experienced ABI during childhood had resources for the transition to adulthood for both healthcare and career interests. Feedback from participants and their families following participation in the B.R.A.I.N program emphasized the importance of addressing health, goal setting, and ensuring a career path based on adolescents’ interests. It is important for adolescents to understand their health needs and to have a medical home that will continue to monitor their health and wellness into adulthood. Having one increases likelihood of a successful transition [20,41]. Education about the effects of brain injuries on their health, life and career pathways is essential to encourage engagement in medical home and support services that facilitate career development. Exercise can improve health and wellness. Adolescents in the current generation have skills in cell phone technology which can assist them with managing issues such as memory independently. Engagement with peer coaches provides mentors to support community engagement and participation in social activities. Collaborative goal setting is crucial for educational and career outcomes. It is particularly important to understand adolescent’s career interests and connect them to resources to achieve a career related to their interests. Discussion of change with physicians, devised plan for changing needs, seeing a doctor who only treats adults should be included in their transition plan [6,20,33,40,42].

There are limitations for this manuscript. We were only able to report on what was done and learned as a program from a clinical perspective. We did not have IRB approval for including specific participant information or outcomes in this manuscript. Although we learned quite a bit, the program was not designed to serve a large number of teens. Over the years, we had more inquires and developed a wait list. Program participants experienced a range of ABI including TBI/Concussion from various mechanisms and brain tumors and also had a range of time since their initial injury which may have contributed to different outcomes following the program.

## 5. Conclusions

Adolescents who experience ABI during childhood benefit from education about the effects of these injuries on their health, wellness and lifestyle and access to services for both healthcare and educational/career transition. Establishing a medical home that continues into adulthood will support monitoring of any persistent symptoms, health, and ensure wellness. Regardless of their educational supports during high school, adolescents benefit from a vocational interest inventory to facilitate understanding of their career interests to support educational and vocational outcomes. Using cell phone technology can support adolescents to manage time and health needs independently. Adolescents and their parents need education on the effects of brain injuries and how this can impact their health and lifestyle after high school to support health management and career development in adulthood. Further research is needed for this adolescent population to better understand how they are doing as adults.

## Figures and Tables

**Table 1:** Program Component Description.

Component	Description
Medical Home	Each participant was asked to describe their medical home, defined as their primary physician and place where parents asked for referrals to specialized physicians.
Goal Setting	We incorporated a Self-determination model into program by asking participants to set goals not only for their long-range plans but also for the skill sets of communication, school, fitness and vocational goals. The book, *Seven Habits of Highly Effective Teens* (Covey) was used to provide lessons in building habits of self- management and organizations. The peer coaches taught the 7 habits over the course of the program in a daily lesson. They then reminded participants to use these ideas as part of their work in the program.
Cell Phone Technology	Cell phones have features to reduce memory load for individuals. Features such as alarms, insertion of repeated entries, and customization of a visual display to suit individual needs offer assistance to those with memory and organization problems for maintaining independence. Contrary to adults, teens in the current generation are technology savvy and are considered to be “technology natives”. Many children are using technology such as word processors and Power Point presentations beginning in second grade. Teens entering rehabilitation programs are likely to have cell phones provided by their parents. However, they tend to use the devices for social networking rather than to improve cognitive performance. We incorporated evidence and our experience and feedback from into our program by working with teens to use cell phone and Smartphone features for self-management of their appointments and assignments. Initially, we received funding from a seed grant from the Center for Pediatric Healthcare Technology Innovation, a joint venture between Children’s Healthcare and Georgia Institute of Technology. As part of the program, we provided participants with an Ipod touch with software for time management, note taking and applications for fitness. We selected the Ipod touch because two school systems in metro Atlanta are currently using this tool to teach academic content. Peer coaches already in the program for communication skills, provided training on device use and delivered experimental tasks for time management. Following the first year, we learned that teens work best with their personal technology provided by their parents. We then proceeded to request that participants bring their own cell phones to the program and utilized peer coaches to service as examples for cell phone use for self-management and organization. During the first day of the program, peer coaches and teens met and discussed how to use the calendar and other features to keep organized and remember what is requested for completion during their time in the program.
Communication Skills	Behavioral and social changes following ABI are linked to outcomes in educational, vocational and community participation [36] Particularly, communication skills, such as initiation and maintaining a conversation, telling information and following a conversation. A reciprocal relationship between communication skills and peer acceptance is reported, which individuals who have ineffective communication skills are likely to be rejected by peers further reducing opportunities for peer interaction. In fact, due to the cognitive and behavioral changes, children and youth with ABI report a gradual loss of friends and increasing loneliness [37,38]. Researchers from several different populations, including young children with developmental disabilities [39] and adults with TBI [38,39] report a positive effect from the training of communication partners. We used this notion in the program to create a “communication–rich” environment for participants. Peer coaches, undergraduates who interviewed from local universities, were trained to create a routine for communication and provide supports for communication skills, based on a model of communication interaction [37,38,39]. Peer coaches received training and practice for 4 strategies: (1) Observe, Wait and Listen (OWL)—Give the adolescent the tools and time to give information without demands, (2) Support a conversation—give information, ask positive questions and provide memory supports, (3) Keep a conversation going—expand, interpret and highlight what the teen says and (4) Help the teen develop conversational skills-be a “supermodel”—keep it clear, fill in the blanks, think out loud, provide opportunities for practice.
Education	During the program we offered two aspects related to education. For participants enrolled in special education, the teacher in the program reviewed the current IEP or 504 plan to determine the type of educational program and goals for the students and if they aligned with the student’s pre- and post- injury level of function and vocational interest inventory(STRONG Vocational Interest Inventory). For participants not enrolled in special education, the teacher reviewed grades, pre- and post- injury level of functioning and met with the participant and their parent to determine if additional accommodations or services were needed.
Fitness and Wellness	Based on feedback from program participants, we added a personal athletic trainer to our program. Each participant received a baseline fitness assessment and set goals for fitness improvement. During the program, peer coaches and volunteers provided classes on ZUMBA, Yoga and dance aerobics to provide the participants with ideas for keeping active.
Leisure activities	Each participant completed a Leisure Survey checklist followed by a discussion with a Therapeutic Recreational Specialist who identified specific preferences and needs for their community. Volunteers in the program created a list of recreational options in the participant’s home community for the participant at discharge.
Driving	We introduced the steps in the Rehabilitation Driving Process that included parental permission, medical clearance, reaction time assessment, written driving test and practice with a driving simulator. Each participant was able to try the driving simulator and received resources for completing a rehabilitation-driving program.

**Table 2:** Examples of Participant and Family report.

Participant	Follow-Up Call Report
MG	My son was doing well but all of a sudden, I received a call-he drove his car into a lake to try to take his life. He was living on his own but after this experience he moved back in with us.
JT	My son tried to get into a medical profession in college, but he did not do well in the courses. He is now working in my husband’s company and doing well. He lives in his own apartment.
SM	My son is still in school, trying to finish his degree in technology. He keeps persisting. He is living with us and receives social security disability.
HG	My daughter is living on her own. She just graduated with her college degree and is applying to Master’s degree programs.

## Data Availability

No new data were created or analyzed in this study. Data sharing is not applicable to this article.
